# Effects of Vitamin D and K on Interleukin-6 in COVID-19

**DOI:** 10.3389/fnut.2021.761191

**Published:** 2022-01-17

**Authors:** Margot P. J. Visser, Anton S. M. Dofferhoff, Jody M. W. van den Ouweland, Henny van Daal, Cornelis Kramers, Leon J. Schurgers, Rob Janssen, Jona Walk

**Affiliations:** ^1^Department of Pulmonary Medicine, Canisius-Wilhelmina Hospital, Nijmegen, Netherlands; ^2^Department of Internal Medicine, Canisius-Wilhelmina Hospital, Nijmegen, Netherlands; ^3^Department of Clinical Chemistry, Canisius-Wilhelmina Hospital, Nijmegen, Netherlands; ^4^Department of Internal Medicine, Radboud University Medical Centre, Nijmegen, Netherlands; ^5^Department of Biochemistry, Cardiovascular Research Institute Maastricht, University of Maastricht, Maastricht, Netherlands

**Keywords:** COVID-19, desmosine, dp-ucMGP, vitamin D, vitamin K, IL-6, 25-hydroxyvitamin D

## Abstract

**Background:**

Pathology during COVID-19 infection arises partly from an excessive inflammatory response with a key role for interleukin (IL)-6. Both vitamin D and K have been proposed as potential modulators of this process.

**Methods:**

We assessed vitamin D and K status by measuring circulating 25-hydroxyvitamin D (25(OH)D) and desphospho-uncarboxylated Matrix Gla-Protein (dp-ucMGP), respectively in 135 hospitalized COVID-19 patients in relation to inflammatory response, elastic fiber degradation and clinical outcomes.

**Results:**

Comparing good and poor disease outcomes of COVID-19 patients, vitamin 25(OH)D levels were not significantly different. IL-6 levels, however, were significantly higher in patients with poor outcome, compared to patients with good outcome (30.3 vs. 153.0 pg/mL; *p* < 0.0001). Dp-ucMGP levels as biomarker of extrahepatic vitamin K status was associated with IL-6 levels (r = 0.35; *p* < 0.0001). In contrast, 25(OH)D levels were only borderline statistically significant correlated with IL-6 (r = −0.14; *p* <0.050). A significant association was also found between IL-6 and elastic fiber degradation. Contrary to vitamin K status, 25(OH)D did not correlate with elastic fiber degradation.

**Conclusions:**

Dp-ucMGP associates with IL-6 as a central component of the destructive inflammatory processes in COVID-19. An intervention trial may provide insight whether vitamin K administration, either or not in combination with vitamin D, improves clinical outcome of COVID-19.

## Introduction

Coronavirus disease (COVID)-19 remains a major global health problem. Pathology during severe acute respiratory syndrome coronavirus 2 (SARS-CoV-2) infection appears to arise at least in part from an excessive inflammatory response with a key role for the cytokine interleukin (IL)-6, which is consistently upregulated in severe COVID-19 (1–3). During SARS-CoV-2 pneumonia, IL-6 production is thought to originate primarily from the pulmonary compartment. This inflammatory response in the lungs leads to immune cell infiltration (4, 5), resulting in damage to pulmonary and vascular structures as well as coagulopathy (6). The subsequent tendency toward hypercoagulability predisposes to arterial, microvascular and venous thrombosis (6). Besides vaccination and general preventative measures to control the pandemic, there is a need for new interventions to reduce disease severity. Both vitamin D and vitamin K have been proposed as treatments that may ameliorate these IL-6-induced pathogenic processes.

By far most research to date has been performed on vitamin D, which is thought to dampen both innate and adaptive immune responses, potentially reducing COVID-19 severity. Vitamin D has been demonstrated to downregulate several cytokines, including IL-6 (7, 8), and, before the emergence of SARS-CoV-2, administration of vitamin D was shown to be protective against acute respiratory tract infections (9). However, studies examining the potential role of vitamin D in COVID-19 show contradictory results (10, 11). Vitamin D is mostly endogenously synthesized in the skin during exposure to sunlight but can be exogenously absorbed from food and supplements as well. Circulating 25-hydroxyvitamin D (25(OH)D) is seen as the best indicator of vitamin D status, since it reflects both intake and production.

Vitamin K was also identified as a candidate modulator of COVID-19 severity (12) based on the strong association between vitamin K insufficiency and poor clinical outcome in two independent cohorts of hospitalized COVID-19 patients (13, 14). It has been proposed that vitamin K-dependent activation of matrix Gla protein (MGP), a potent protector of soft tissues against various insults such as mineralization and degradation, is critical for dampening inflammation-induced damage to vascular and pulmonary tissues during SARS CoV-2 infection (12). However, vitamin K may also suppress IL-6 production, both indirectly through its activation of the immune inhibitory proteins growth-arrest-specific gene 6 (Gas6) (15, 16) and protein S (17, 18), or directly by inhibiting phosphorylation of IKKα/β that is required for activation of nuclear factor (NF)κB (19, 20).

Vitamin K status might be assessed by several methods. The indirect measurement of surrogate markers of vitamin K deficiency such as inactive MGP is considered valid and representative of vitamin K status. Inactive MGP reflects the combined extrahepatic deficit of vitamin K1 and K2 (21, 22).

In the present study we examined the associations of both vitamin D and K status with the inflammatory responses, elastic fiber degradation and clinical outcome of hospitalized COVID-19 patients.

## Materials and Methods

### Subjects and Blood Sampling

Both vitamin D and K status, IL-6 levels and the rate of elastic fiber degradation were quantified in blood samples of 135 consecutive hospitalized COVID-19 patients in the Canisius-Wilhelmina Hospital between March 12^th^ and April 15^th^ 2020. The primary reason for hospital admission had to be COVID-19 whereby SARS-CoV-2 infection was confirmed by Real Time polymerase chain-reaction testing. Patients admitted for other reasons with coincidentally positive PCR tests were excluded. Both male and female individuals over 18 years old were included. Individuals using vitamin K antagonists were included as well. Patients were informed about the study and could opt-out if requested. Data was extracted from hospital electronic patient records. The majority of patients were treated with (hydroxy)chloroquine and prophylactic heparin-based anticoagulants (low molecular weight heparin, unless already taking oral anticoagulants). Tocilizumab and dexamethasone were not administered in the first Dutch COVID-19 wave. From each patient, blood was sampled in EDTA tubes, aliquotted and frozen at−80 degrees Celsius. Follow-up of COVID-19 patients took place until (1) discharge from the hospital, (2) intubation and invasive mechanical ventilation, or (3) death. Disease outcome was categorized as ‘good' when patients were discharged from the hospital without the need for mechanical ventilation, and ‘poor' if patients required intubation and invasive ventilation and/or died.

### Measurement of Interleukin-6

IL-6 levels were determined in EDTA plasma samples using the commercially available Elecsys IL-6 immunoassay on a Cobas e immunoassay analyzer (Roche Diagnostics, Switzerland), according to manufacturer's instructions. This assay is currently being validated as a predictor of disease progression in COVID-19 and has been granted a US Food and Drug Administration's Emergency Use Authorization (https://diagnostics.roche.com/us/en/products/params/elecsys-il-6.html). The within-run and between-run precision are 1.9–10.3% and 4.1–11.7%, respectively. Its measuring range is between 1.5–5,000 pg/mL. Normal range is up to 7 pg/ml.

### Dp-ucMGP as Measurement of Extrahepatic Vitamin K Status

Plasma dp-ucMGP levels were determined using the commercially available CE-marked IVD chemiluminescent InaKif MGP assay on the IDS-iSYS system (IDS, Boldon, UK) as previously described (23). The within-run and total modulation of this assay were 0.8–6.2% and 3.0–8.2%, respectively.

The assay measuring range was between 200–12,000 pmol/L and linear up to 11,651 pmol/L. Dp-ucMGP levels <300 pmol/L are in the normal healthy range and levels >500 pmol/L reflect vitamin K insufficiency (24).

### Measurement of 25-Hydroxyvitamin D

25(OH)D levels were measured in EDTA plasma using a validated liquid chromatography-tandem mass spectrometry (LC-MS/MS) as previously described (25). Vitamin D deficiency was defined as a 25(OH)D concentrations <25 nmol/L, insufficiency between 25 and 50 nmol/L, and sufficiency ≥50 nmol/L.

### Measurement of Desmosine

Elastic fibers are fundamental matrix components in lungs. Desmosine and isodesmosine (DES) are amino acids that are unique to cross-linked elastic fibers and are released in the blood stream after degradation. DES was therefore used as surrogate biomarker for the rate of elastic fiber degradation. DES fractions were measured using Liquid Chromatography with tandem mass spectrometry (LC-MS/MS) as previously described (13). Coefficient of variations of intra- and inter-assay imprecision were <8.2%, lower limit of quantification of 140 ng/L, and assay linearity up to 210,000 ng/L.

### Statistical Analysis

Statistical analyses were performed using SPSS (version 24, IBM SPSS Statistics for Windows, Europe) and GraphPad Prism 5 (version 5.03 for Windows). As IL6, dp-ucMGP, 25(OH)D and DES values were not normally distributed, statistical analyses was done by using Mann-Whitney U test to compare means between two groups and Kruskal-Wallis test to compare means between multiple groups. Correlations were assessed over log-transformed data using Pearson coefficient. Both uni- and multivariable linear regression analyses were used to compare dp-ucMGP, vitamin (25-OH)D and desmosine levels with IL-6 levels, adjusted for age and gender. Uni- and multivariate logistic regression analyses were used to compare dp-ucMGP, vitamin (25-OH)D, desmosine and IL-6 levels with disease outcome, adjusted for age and gender. As threshold for statistical significance, a *p*-value of <0.050 was used.

## Results

In total, 137 patients were consecutive included in the study, meeting the inclusion criteria. Two patients opt out after informing. Baseline characteristics and outcome are shown in Table 1.

**Table 1:** Baseline characteristics and outcome of all 135 individuals.

**Characteristic**	**Subjects *N* (%)**
Age (years)	68 ± 12
Male (%)	93 (69)
VKA use (%)	12 (8.9)
Dialysis dependent (%)^*^	3 (2.2)
Immunocompromised (%)	6 (4.4)
Respiratory disease (%)	40 (29.6)
Cardiac or cardiovascular disease (%)	38 (28.1)
**Laboratory parameters**	**N (Median; LQ-UQ)**
IL-6, pg/mL	133 (57; 26–153)
Desmosine, ng/L	127 (353; 280–491)
Dp-ucMGP, pmol/L	135 (1137.7; 816–1676)
Vitamin (25-OH)D, nmol/L	133 (40.4; 27–66)
**Outcome**	***N*** **(%)**
ICU admission (%)	36 (26.7)
ICU admission and mechanical ventilation (%)	32 (23.7)
Deceased (%)	42 (31.1)
Poor outcome (%)	60 (44.4)

*VKA, Vitamin K antagonist*.

* *At admission*.

### IL-6 Levels, Elastic Fiber Degradation and Clinical Outcome

**Figure 1:**
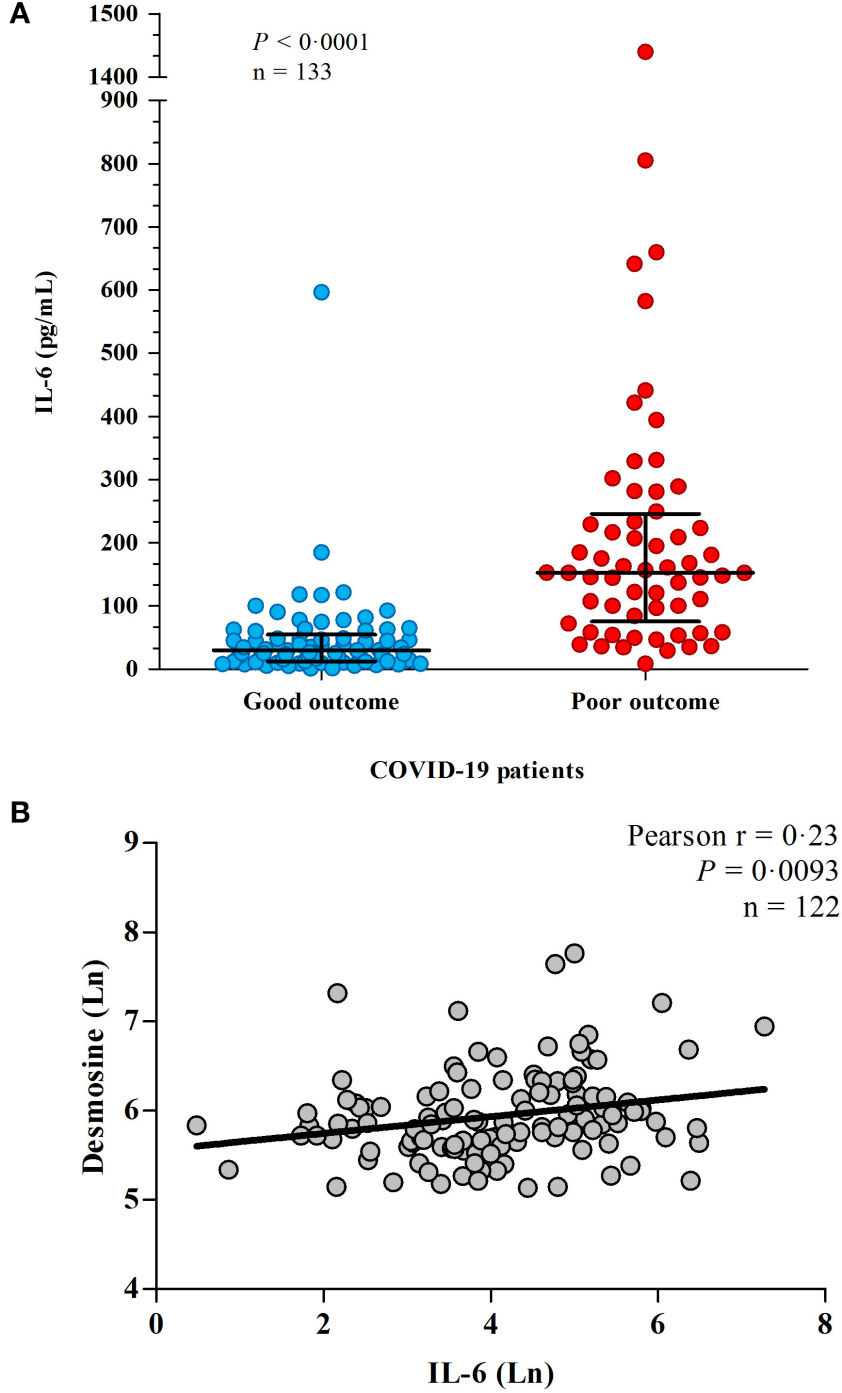
**(A)** IL-6 levels in hospitalized patients with COVID-19. IL-6 levels were measured in plasma from 133 patients. Patients outcome was defined as “good” when they survived without the need of invasive ventilation, or “poor” when they needed supportive invasive ventilation and/or deceased. **(B)** The association between IL-6 and elastic fiber degradation. Correlation between IL-6 and desmosine in 122 COVID-19 patients, both log transformed. Line shows the result of a linear regression.

Plasma IL-6 was measured in 133 hospitalized COVID-19 patients. The mean age of hospitalized COVID-19 patients was 68 ± 12 years and 93 (69%) were male. At admission, circulating IL-6 concentrations were significantly higher in patients with poor clinical outcomes than in those with good outcome (median 30.32 pg/mL interquartile range 12.73–54.60 pg/mL vs. median 153.0 pg/mL interquartile range 75.53–245.7 pg/mL; *p* < 0.0001; Figure 1A), which remained significant after adjustment for age and gender (*p* ≤ 0.0001).

Desmosine crosslinks elastic fibers and its presence in the circulation is a robust biomarker for elastic fiber damage (26). We previously described a correlation between elevated DES levels and poor clinical outcome (13). DES was successfully measured in 125 patients. Three patients who were hemodialysis dependent due to kidney failure, which existed before hospital admission, were excluded from this analysis since plasma DES is strongly dialyzed (R. Janssen, unpublished data). Only blood samples before initiation of renal replacement therapy were used of patients who developed an indication for this treatment during hospital admission. IL-6 levels strongly correlated with plasma desmosine (*p* = 0.0017; Figure 1B), which remained significant after adjustment for age and gender (*p* = 0.023), suggesting a key link between inflammation and pulmonary/vascular tissue damage in COVID-19.

### Vitamin D Status, Elastic Fiber Degradation and Clinical Outcome

We previously demonstrated that extrahepatic vitamin K status insufficiency correlated with poor clinical outcome as well as accelerated elastic fiber degradation in this cohort (13). Contrary to vitamin K status, 25(OH)D levels were comparable between patients with good and poor outcomes, with no difference in 25(OH)D levels (median 45.00 nmol/L interquartile range 26.71–67.90 nmol/L vs. 37.65 nmol/L interquartile range 26.93–63.19 nmol/L; *p* = 0.85), results remained unchanged after adjustment for age and gender (*p* = 0.27)

**Figure 2:**
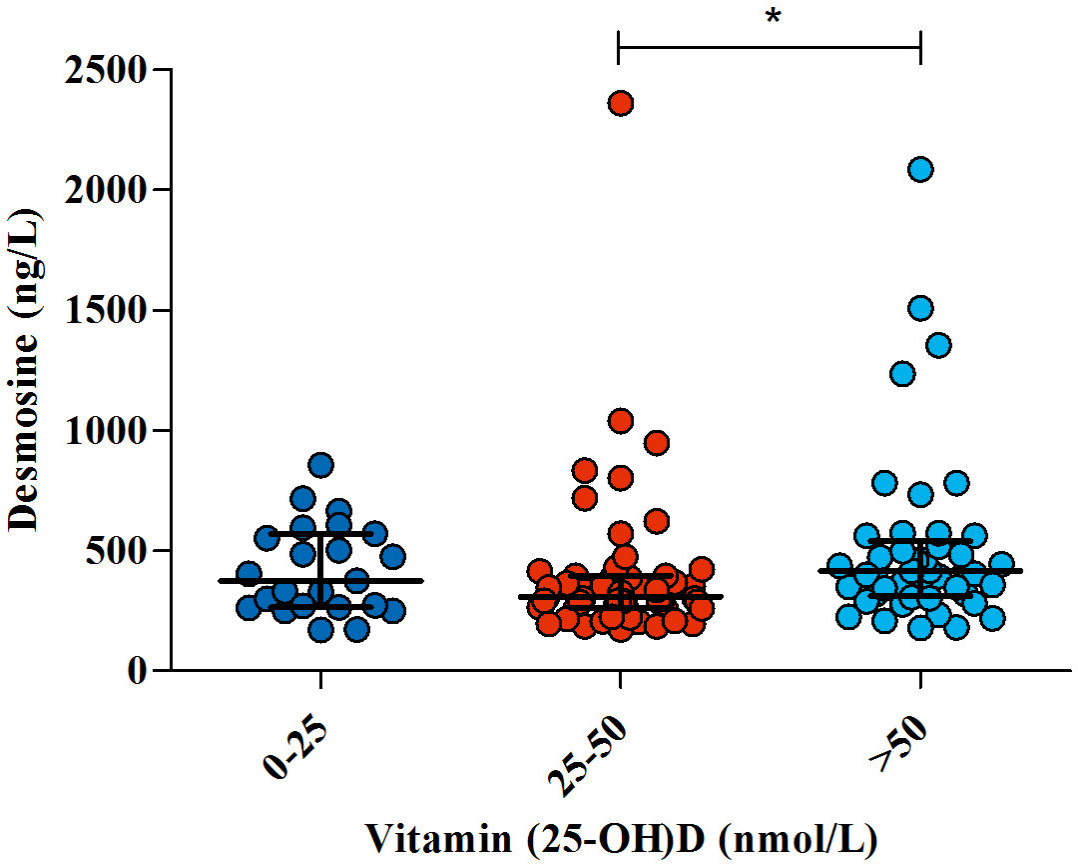
The association between 25(OH) vitamin D status and elastic fiber degradation. In 122 COVID-19 patients, 25(OH) vitamin D and desmosine were measured as a marker of elastic fiber degradation. Three hemodialysis patients were excluded from the analysis. Patients were split up based on vitamin D status. Lines and error bars represent median and interquartile range. ^*^Significant difference was found in desmosine levels in patients with vitamin D insufficiency compared to sufficient patients.

Similarly, there was no correlation between 25-(OH)D levels and plasma DES (*p* = 0.22), neither after adjustment for age and gender (*p* = 0.918). Subsequently, patients were separated into three categories based on vitamin D status: (1) vitamin D deficient (<25 nmol/L), (2) vitamin D insufficient (25–50 nmol/L), or (3) vitamin D sufficient (>50 nmol/L). Although there was no overall correlation between 25(OH)D and DES, there was significant difference in DES levels between the groups (*p* = 0.019), with significantly lower DES in patients with vitamin D insufficient compared to sufficient patients, Figure 2.

### Vitamin K and D Status and Inflammation

Dp-ucMGP is an indirect marker of extrahepatic vitamin K status, where high levels of dp-ucMGP indicate vitamin K insufficiency and *vice versa* (21).

**Figure 3:**
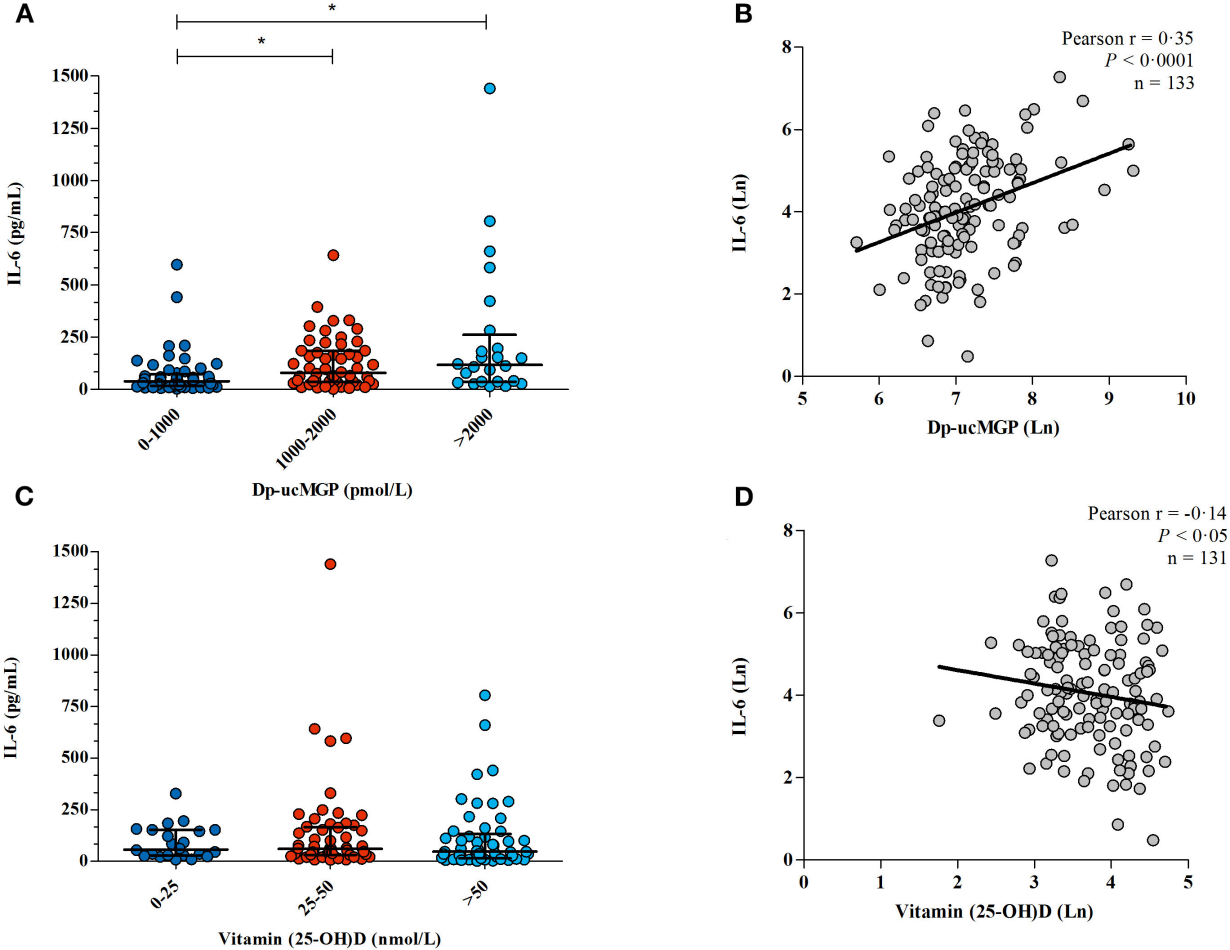
The association between vitamin K /vitamin D and IL-6. **(A)** Dp-ucMGP and IL-6 were measured in 133 COVID-19 patients. Patients were separated into three categories, mild (dp-ucMGP <1,000 pmol/L), moderate (dp-ucMGP between 1,000–2,000 pmol/L) and severe vitamin K deficiency (dp-ucMGP >2,000 pmol/L). *Significant difference in IL-6 levels was found between the dp-ucMGP groups 0–1,000 pmol/L and 1,000–2,000 pmol/L. **(B)** Correlation between IL-6 and dp-ucMGP, both log transformed. Line shows the result of a linear regression. **(C)** Vitamin 25(OH) D and IL-6 were measured in 131 COVID-19 patients. IL-6 levels were compared with different levels of vitamin 25(OH) D. There is no significant difference in IL-6 levels between groups. **(D)** Correlation between IL-6 and vitamin 25(OH) D, both log transformed. Line shows the result of a linear regression.

As previously published (13), all patients in this cohort had at least a mild vitamin K insufficiency (*i.e*. dp-ucMGP >500 pmol/L). Patients with moderate or severe vitamin K deficiency had significantly higher levels of IL-6 compared to those with mild vitamin K deficiency (median 78.11 pg/mL interquartile range 35.36–185.0 pg/mL and median 116.2 pg/mL interquartile range 36.64–260.5 pg/mL vs. median 39.11 interquartile range 17.00-72.43 pg/mL; *p* = 0.0004; Figure 3A), and dp-ucMGP levels were significantly correlated with IL-6 (Pearson r = 0.35; *p* < 0.0001; Figure 3B).

In contrast, there was no significant difference in IL-6 between patients with vitamin D deficiency, insufficiency or sufficiency (*p* =‘0.47; Figure 3C), though there was a borderline statistically significant correlation between higher vitamin D levels and lower IL-6 concentration(Pearson r = −0.14; *p* <0.050 Figure 3D).

### Univariable and Multivariable Linear and Logistic Regression Analyses

At multivariable analysis, most important independent factors associated with IL-6 levels were sequentially disease outcome (*p* < 0.001), dp-ucMGP (*p* = 0.004) and gender (*p* = 0.024) (Table 2). In contrast to the univariable analysis, desmosine was not of significant impact on IL-6 levels, when adjusted for other risk factors (Table 2). At univariable analysis of disease outcome, independent variables age, dp-ucMGP, desmosine and IL-6 were turned out to be associated. Regarding to the multivariable analysis, desmosine and IL-6 levels had the strongest association.

**Table 2:** Univariable and multivariable analyses assessing factors associated to IL-6 levels and disease outcome.

	**IL-6**				**Disease outcome**			
	**Univariable**		**Multivariable**		**Univariable**		**Multivariable**	
**Variable**	**B (SE)**	***P***	**B (SE)**	***P***	**B (SE)**	***P***	**B (SE)**	***P***
Age	0.019 (0.009)	0.031	−0.008 (0.008)	0.334	0.065 (0.017)	<0.001	0.038 (0.029)	0.189
Gender	0.780 (0.228)	0.001	0.436 (0.191)	0.024	0.824 (0.393)	0.036	0.348 (0.608)	0.568
Dp-ucMGP	0.719 (0.169)	<0.001	0.534 (0.181)	0.004	0.960 (0.328)	0.003	−0.386 (0.545)	0.480
Vitamin (25-OH)D	−0.325 (0.196)	0.100	−0.193 (0.159)	0.229	−0.137 (0.309)	0.658	−0.345 (0.474)	0.467
Desmosine	0.587 (0.222)	0.009	−0.118 (0.232)	0.610	1.895 (0.489)	<0.001	1.554 (0.777)	0.045
IL-6	-	-	-	-	1.811 (0.309)	<0.001	1.669 (0.327)	<0.001
Outcome	1.623 (0.170)	<0.001	1.433 (0.196)	<0.001	-	-	-	-

## Discussion

Both vitamin D and vitamin K have been suggested as disease modifiers of SARS-CoV-2 infection. We evaluated the association between IL-6 levels in hospitalized COVID-19 patients and 25(OH)D and dp-ucMGP as measures of vitamin D and vitamin K status, respectively. We found that dp-ucMGP was correlated with circulating IL-6 levels, even after adjustment for other risk factors. The association between 25(OH)D and IL-6 was weaker, though borderline statistically significant. In contrast to extrahepatic vitamin K status (13), higher vitamin D levels were not associated with better clinical outcome.

Elevated IL-6 has been a consistent finding in severe COVID-19 (1). This led many to make a comparison with ‘cytokine storm', as is seen in sepsis or in response to chimeric antigen receptor (CAR) T cell therapy (27). However, it was recently pointed out that the term ‘cytokine storm' might not encompass the entirety of the pathological mechanisms underlying severe COVID-19, which also includes endovasculitis, direct pulmonary injury, coagulopathy and immunosuppression (28). Furthermore, abrogating IL-6 signaling through receptor blockade with monoclonal antibodies has produced mixed results and offers at most a modest improvement of outcome (29, 30), while removing circulating cytokines during extracorporeal membrane oxygenation (ECMO) was even associated with increased mortality (31). These somewhat surprising findings may be explained by the pleiotropic nature of IL-6, which besides its well-known pro-inflammatory functions also has key anti-inflammatory properties and is crucial for cell regeneration (30).

Our findings support the case for a net negative effect of IL-6 in COVID-19 as we demonstrate that DES, a downstream biomarker of tissue damage, associates with higher circulating IL-6. However, where monoclonal antibodies non-selectively block IL-6 signaling and may consequently be expected to have both favorable and unfavorable effects, we hypothesize that IL-6 modulation by vitamin K may instead be more selectively beneficial (32).

There are various mechanisms through which vitamin K may influence the inflammatory course of SARS-CoV-2 infection. Protein S and Gas6 specifically dampen monocyte and macrophage production of IL-6 through their binding to the Tyro3/Axl/Mer (TAM) family of receptor tyrosine kinases (15, 18). This binding requires vitamin K-dependent carboxylation. It has already been suggested that loss of protein S is associated with a loss of crucial immune inhibition that has detrimental effects on COVID-19 severity (18, 33). Similarly, vitamin K is thought to directly dampen NFκB signaling, one of the pathways involved in IL-6 expression, which has been implicated in macrophages (34, 35) and pulmonary epithelium in COVID-19 (36). This might result in a more specific IL-6 inhibition than achieved through administration of tocilizumab (37). Furthermore, in contrast to treatments that solely interfere with IL-6, vitamin K not only modulates inflammation but also targets other causes of SARS-CoV-2 induced pathology, including vascular and pulmonary damage as well as coagulopathy. In this study, both IL-6 and dp-ucMGP positively associate with DES, which supports the hypothesis that vitamin K deficiency relates to the unfavorable effects of IL-6.

Approximately 50% of patients in our cohort also had vitamin D insufficiency or deficiency, similar to the general incidence in The Netherlands (38). Population studies have suggested a link between vitamin D deficiency and susceptibility to COVID-19 (39, 40). Although only interventional studies can provide definitive answers on causality, in our cohort vitamin D sufficiency was not associated with better clinical outcome.

This difference may lie in contrasting effects of vitamin D on COVID-19 ‘severity' and ‘susceptibility', with a critical clue provided by the observation that vitamin D sufficiency was associated with higher levels of elastic fiber damage compared to a mild insufficiency. We theorize that vitamin D might act like a double-edged sword in the COVID-19 pathogenesis, with potential positive and negative effects. On the one hand, vitamin D has a slight dampening effect on IL-6. On the other hand, it plays a role in calcium metabolism and contributes to a (transient) increase in serum calcium (41). Elastic fibers have high calcium affinity, especially when they have been partially degraded by proteases as may occur during COVID-19-associated inflammation (42). Resulting calcification further stimulates elastic fiber degradation (43, 44), which might explain the accelerating effect of vitamin D in this process. Calcium binding to elastic fibers is expected to persist after correction of circulating calcium levels, whereas systemic calcium is under strict homeostatic control (45). Through these mechanisms vitamin D may have a favorable role on IL-6 but a detrimental effect on elastic fiber metabolism, resulting in a net neutral effect on disease severity and clinical outcome. This would be in line with a previous study in which vitamin D administration increased circulating DES in vitamin D deficient patients with chronic obstructive pulmonary disease (46).

**Figure 4:**
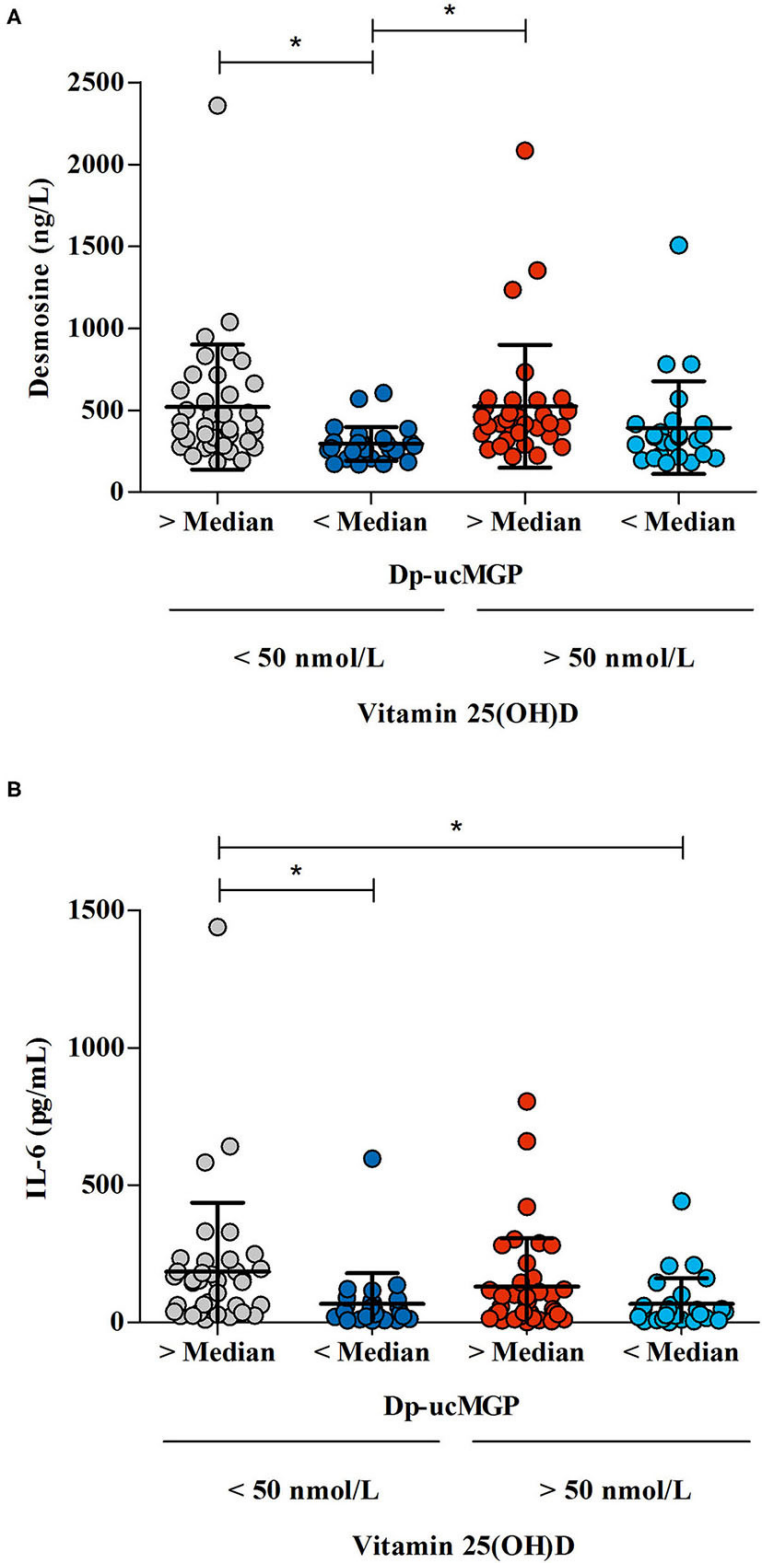
The association between the differences in vitamin K status /vitamin D status /desmosine and IL-6 levels in COVID-19 patients. Vitamin K levels were defined as “low” when dp-ucMGP levels were above median, and “high” when dp-ucMGP levels were below median. Vitamin D levels were defined as “low” when there was a 25(OH)D insufficiency (concentration < 50 nmol/L) and “high” when there was a sufficient amount of vitamin D (concentration >50 nmol/L). **(A)** The effect of vitamin K status — derived from dp-ucMGP status — on desmosine levels in patients with high or low vitamin D levels. Desmosine levels were measured in 122 patients. **(B)** The effect of vitamin K status — derived from dp-ucMGP status — on IL-6 levels in patients with high or low vitamin D levels (*n* = 131). ^*^Indicates significant difference between groups.

The effects of vitamin D should also be placed in context of the fact that all our patients were vitamin K deficient. Medication administered during hospitalization may have potential effects on vitamin D status. It is found that hydroxychloroquine prevents vitamin D deficiency in patients with systemic lupus erythematosus. It is suggested that this may be a result of limited conversion of 25(OH)D to 1,25(OH)_2_D (47). In contrast, low molecular weight heparins possibly adversely affect vitamin D metabolism (47). However, there is a low probability of any affect during the short time period these drugs were prescribed. Administration of high doses of vitamin D in rats induced rapid and severe calcification of the lungs and upregulation of pulmonary MGP expression (48). In kidney transplant patients, a population in which vascular calcification largely contributes to morbidity and mortality, vitamin D supplementation actually increased mortality in vitamin K deficient patients (49). This may be reasonable considering the significance of vitamin K-activated MGP in protecting against elastic fiber calcification and degradation (48). It can be hypothesized that vitamin D supplementation puts an excessive burden on already depleted vitamin K stores during COVID-19, with subsequent destructive consequences for elastic fibers (Figure 4) (48).

However, this may present a therapeutic opportunity, if vitamin D is administered after any vitamin K deficiency is corrected, this could allow for the positive benefits of vitamin D without excessive damage due to calcification of elastic fibers.

A limitation of the current study is that it was conducted in one region in a single month, while vitamin D levels vary strongly between populations and throughout the year. Therefore, it will be important to validate these findings in other cohorts. However, there was significant variability in vitamin D status between our patients, strengthening our findings. The current study is limited by its observational nature. We chose to focus our study on IL-6 as one of the most established correlates of Covid-19 disease severity and a current therapeutic target, and did not assess the relationship between vitamin K and D status and other clinical laboratory parameters such as CRP or other pro-inflammatory cytokines like TNF-α.

Clinical intervention studies will be required to establish whether supplementation of vitamin K, possibly in combination with vitamin D, has a positive effect on COVID-19 progression. Due to the extremely high prevalence of pronounced vitamin K deficiency in severe COVID-19 patients and its strong association with increased inflammation, elastic fiber degradation and poor clinical outcome, such studies should be prioritized given the on-going COVID-19 pandemic.

## Data Availability Statement

The dataset used during the current study is available from the corresponding author upon reasonable request.

## Ethics Statement

This study involving human participants was reviewed and approved by Ethical Review Board CMO Arnhem-Nijmegen, which waived the need for written informed consent. Written informed consent was not required for the study on human participants in accordance with the local legislation and institutional requirements. Patients provided verbal consent and could opt-out after they were informed about the study.

## Author Contributions

RJ, AD, and JW designed the study. LS was responsible for the dp-ucMGP. JO and HD were responsible for the DES, 25(OH)D, and IL-6 measurements. MV analyzed the data and performed statistical analysis. MV, RJ, and JW wrote the first draft of the manuscript. AD, JO, HD, CK, and LS critically revised the manuscript. All authors contributed to the article and approved the submitted version.

## Funding

Kappa Bioscience AS contributed with a PhD grant to vitamin K2 related COVID-19 research at Department of Internal Medicine, Canisius-Wilhelmina Hospital.

## Conflict of Interest

RJ discloses application of a patent on vitamin K in COVID-19. MV, RJ, JW, and AD have a scientific collaboration with Kappa Bioscience AS, a manufacturer of vitamin K2 (MK-7). JO and RJ are owners of (Desmosine.com). LS received research funding not related to this work and is stockholder in Coagulation Profile. The remaining authors declare that the research was conducted in the absence of any commercial or financial relationships that could be construed as a potential conflict of interest.

## Publisher's Note

All claims expressed in this article are solely those of the authors and do not necessarily represent those of their affiliated organizations, or those of the publisher, the editors and the reviewers. Any product that may be evaluated in this article, or claim that may be made by its manufacturer, is not guaranteed or endorsed by the publisher.
