# Corrigendum: Effects of Vitamin D and K on Interleukin-6 in COVID-19

**DOI:** 10.3389/fnut.2022.868324

**Published:** 2022-03-08

**Authors:** Margot P. J. Visser, Anton S. M. Dofferhoff, Jody M. W. van den Ouweland, Henny van Daal, Cornelis Kramers, Leon J. Schurgers, Rob Janssen, Jona Walk

**Affiliations:** ^1^Department of Pulmonary Medicine, Canisius-Wilhelmina Hospital, Nijmegen, Netherlands; ^2^Department of Internal Medicine, Canisius-Wilhelmina Hospital, Nijmegen, Netherlands; ^3^Department of Clinical Chemistry, Canisius-Wilhelmina Hospital, Nijmegen, Netherlands; ^4^Department of Internal Medicine, Radboud University Medical Centre, Nijmegen, Netherlands; ^5^Department of Biochemistry, Cardiovascular Research Institute Maastricht, University of Maastricht, Maastricht, Netherlands

**Keywords:** COVID-19, desmosine, dp-ucMGP, vitamin D, vitamin K, IL-6, 25-hydroxyvitamin D

In the original article, there was a mistake in Figure 4 as published. The </> signs were accidentally switched in the figure. The corrected Figure 4 appears below.

**Figure 4:**
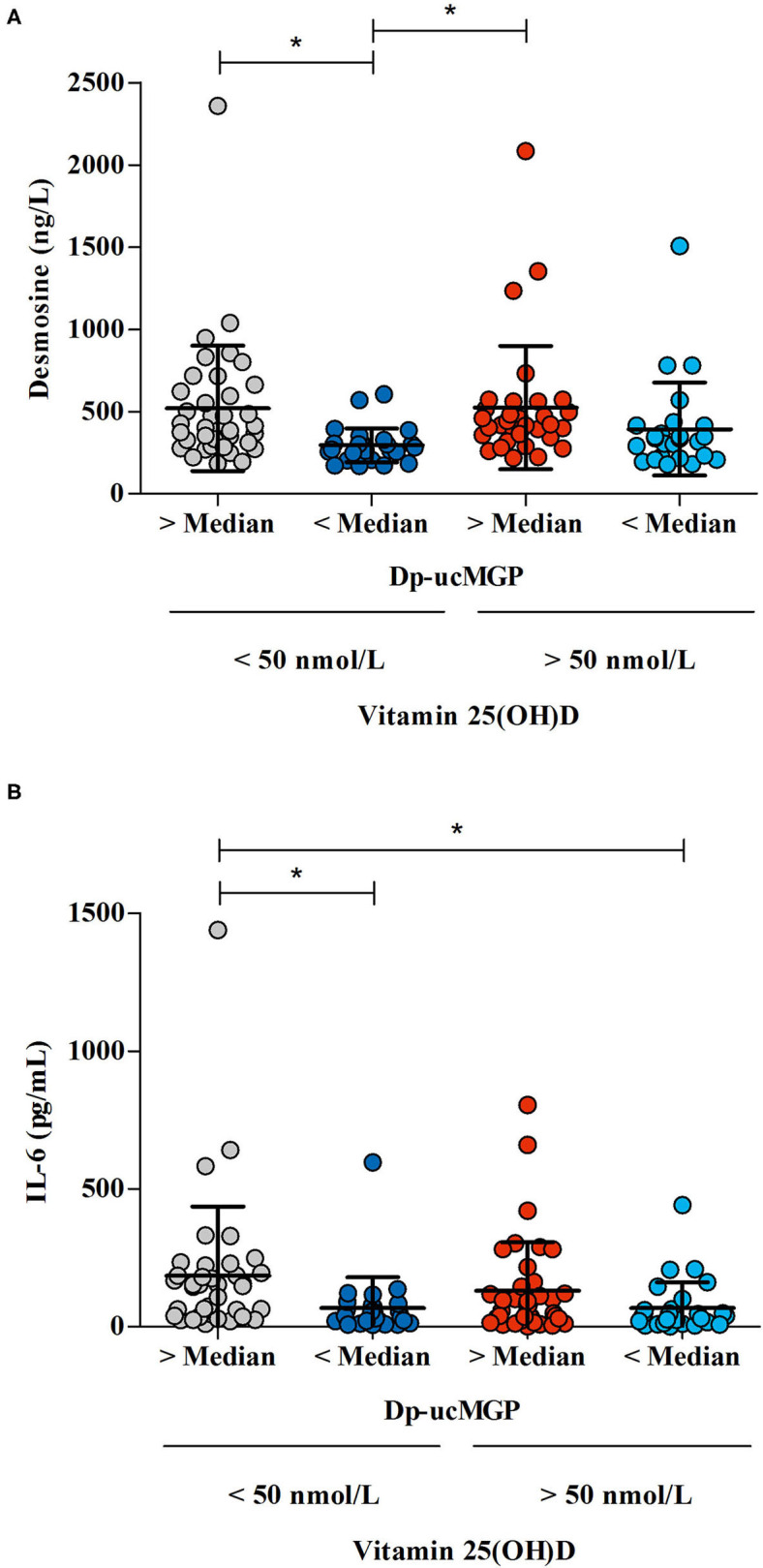
The association between the differences in vitamin K status /vitamin D status /desmosine and IL-6 levels in COVID-19 patients. Vitamin K levels were defined as “low” when dp-ucMGP levels were above median, and “high” when dp-ucMGP levels were below median. Vitamin D levels were defined as “low” when there was a 25(OH)D insufficiency (concentration <50 nmol/L) and “high” when there was a sufficient amount of vitamin D (concentration >50 nmol/L). **(A)** The effect of vitamin K status — derived from dp-ucMGP status — on desmosine levels in patients with high or low vitamin D levels. Desmosine levels were measured in 122 patients. **(B)** The effect of vitamin K status — derived from dp-ucMGP status — on IL-6 levels in patients with high or low vitamin D levels (*n* = 131). ^*^Indicates significant difference between groups.

The authors apologize for this error and state that this does not change the scientific conclusions of the article in any way. The original article has been updated.

## Publisher's Note

All claims expressed in this article are solely those of the authors and do not necessarily represent those of their affiliated organizations, or those of the publisher, the editors and the reviewers. Any product that may be evaluated in this article, or claim that may be made by its manufacturer, is not guaranteed or endorsed by the publisher.

